# Imaging-Based Disease Assessment and Management Recommendations: Impact of Multidisciplinary Sarcoma Tumor Board

**DOI:** 10.3390/cancers16152674

**Published:** 2024-07-26

**Authors:** Maverick Jubane, Andrew C. Rennick, Joseph J. Villavicencio, Felipe Ferreira de Souza, Vanessa Peters, Emily Jonczak, Steven Bialick, Aditi Dhir, Julie Grossman, Jonathan C. Trent, Gina D’Amato, Andrew E. Rosenberg, Francis J. Hornicek, Raphael L. Yechieli, Ty Subhawong, Francesco Alessandrino

**Affiliations:** 1 Department of Radiology, Jackson Memorial Hospital, Miami, FL 33136, USA; 2 Leonard M. Miller School of Medicine, University of Miami, Miami, FL 33136, USA; 3 Department of Radiology, University of Miami, Miami, FL 33136, USA; 4 Department of Interventional Radiology, University of Miami, Miami, FL 33136, USA; 5 Sylvester Comprehensive Cancer Center, Miami, FL 33136, USA; 6 Division of Medical Oncology, Department of Medicine, University of Miami, Miami, FL 33136, USA; 7 Division of Pediatric Hematology & Oncology, Department of Pediatrics, University of Miami, Miami, FL 33136, USA; 8 Division of Surgical Oncology, Department of Surgery, University of Miami, Miami, FL 33136, USA; 9 Department of Pathology & Laboratory Medicine, University of Miami, Miami, FL 33136, USA; 10 Department of Orthopedics, University of Miami, Miami, FL 33136, USA; 11 Department of Radiation Oncology, University of Miami, Miami, FL 33136, USA

**Keywords:** sarcoma, medical oncology, radiology, radiology reports

## Abstract

**Simple Summary:**

Sarcomas are a heterogeneous group of tumors that arise from mesenchymal tissue, including adipose, bone, cartilage, skeletal muscle, and smooth muscle. Because of their rarity, optimal treatment planning requires discussion among sarcoma-experienced subspecialists. Studies have shown that treatment guided by multidisciplinary tumor boards (MTBs) is associated with better compliance with clinical practice guidelines and, in some cases, increased survival. The role of radiologists in the MTB setting is valuable in assessing disease in patients with sarcoma; however, radiologists’ recommendations may differ from the MTB’s consensus. This study highlights the discordance between the disease assessment and the clinical recommendations provided by radiologists and MTBs and encourages all radiologists caring for patients with sarcoma to participate in MTBs to best align their interpretations with optimal clinical management and make multidisciplinary aligned recommendations whenever feasible.

**Abstract:**

Multidisciplinary tumor boards (MTBs) facilitate decision-making among subspecialists in the care of oncology patients, but the mechanisms by which they enhance outcomes remain incompletely understood. Our aim was to measure the agreement between sarcoma MTBs and radiology reports’ disease assessment and management recommendations. This single-center IRB-approved retrospective study evaluated cases presented at a weekly sarcoma MTB from 1 August 2020 to 31 July 2021. Cases without clinical notes, imaging studies, or radiology reports were excluded. The data collected included the patient’s clinical status at the time of the MTB, the treatment response assessment by the MTB and radiologists (stable disease; partial response; complete response; progressive disease/recurrence), and the recommendations of the radiology reports and of the MTB. The agreement between the initial radiologist review and MTB on disease assessment and recommendations was analyzed using kappa statistics. In total, 283 cases met the inclusion criteria. Radiology reports provided recommendations in 34.3% of cases, which were adhered to by the ordering providers in 73.2% of cases. The agreement between MTBs and radiology reports was moderate in disease assessment (86.2% agreement; κ = 0.78; *p* < 0.0001) and negligible in recommendations (36% agreement; κ = 0.18; *p* < 0.0001). Radiologists were more likely to assign progressive disease/recurrence than MTBs (54.4% vs. 44.4%; *p* < 0.001) and to recommend short-term imaging follow-up more commonly than MTBs (46.4% vs. 21.7%; *p* < 0.001). At a tertiary care center, radiologists’ isolated interpretations of imaging findings and management recommendations frequently differ from the MTB’s consensus, reflecting the value of multidisciplinary discussions incorporating the patient’s clinical status and the available treatment options into the final radiographic assessment.

## 1. Introduction

Sarcomas are a heterogeneous group of tumors that arise from mesenchymal tissue, including adipose, bone, cartilage, skeletal muscle, and smooth muscle [1]. Accounting for <1% of all neoplasms, their rarity often results in a delay in diagnosis and in the initiation of potentially life-saving therapy [2]. The most common types of soft tissue sarcoma include undifferentiated pleomorphic sarcoma, myxofibrosarcoma, liposarcoma, and leiomyosarcoma, with factors including the tumor size, histologic grade, and distant disease determining the clinical stage and prognosis [3]. Surgery remains the mainstay of local control, frequently in conjunction with neoadjuvant chemotherapy and radiotherapy [1]. Because of the rarity of the disease, the complexity of care, and the lack of algorithms that apply to all situations, optimal sarcoma treatment planning requires discussion among sarcoma-experienced subspecialists [4]. Studies have shown that treatment guided by multidisciplinary tumor boards (MTBs) is associated with better compliance with clinical practice guidelines and, in some cases, increased survival. In sarcoma specifically, MTBs improve compliance with clinical practice guidelines, including the National Comprehensive Cancer Network guidelines, and increase relapse-free survival in patients with sarcoma when the initial treatment is based on the MTB decision [5,6,7,8]. 

Many academic and community oncology programs utilize specialized MTBs with the aim of improving patient outcomes by guaranteeing appropriate disease care and standardizing treatment strategies [9]. MTBs are frequently composed of both medical and surgical specialists, including surgical oncologists, orthopedic oncologists, medical oncologists, radiation oncologists, pathologists, interventional radiologists, and diagnostic radiologists [10]. The diversity in specialties allows for a thorough review of patient information, comprising the medical and surgical history, initial pathological findings, and prior surgical and adjuvant therapies, as well as a physical review of diagnostic imaging and histopathology, all by experienced subspecialists [10]. Frequently, MTBs utilize their specialists to confirm the histologic subtype and grade and reinterpret radiologic examinations to arrive at a consensus treatment and management plan through shared decision-making. 

In fact, discrepancies between radiologists and second opinion reads during MTBs are often observed, often with a significant impact on patient management, with changes in the primary diagnosis, the assessment of the treatment response, or the treatment plans for various cancers, including sarcomas [11,12,13,14,15]. However, while many studies have focused on assessing the discrepancies in the diagnosis status between the radiology report and the MTB, few focus on evaluating the discrepancies between the management recommendations by the MTB and the radiology report [12,14,16,17]. Given the highly variable rate at which the radiology report recommendations are followed by the referring providers, it is worth exploring whether the radiology report recommendations differ from the recommendations of a subspecialty-focused multidisciplinary team [18,19]. As such, we aimed to measure the agreement between the MTB and the radiology reports’ management recommendations and the disease assessment.

## 2. Materials and Methods

### 2.1. Study Population

**Figure 1:**
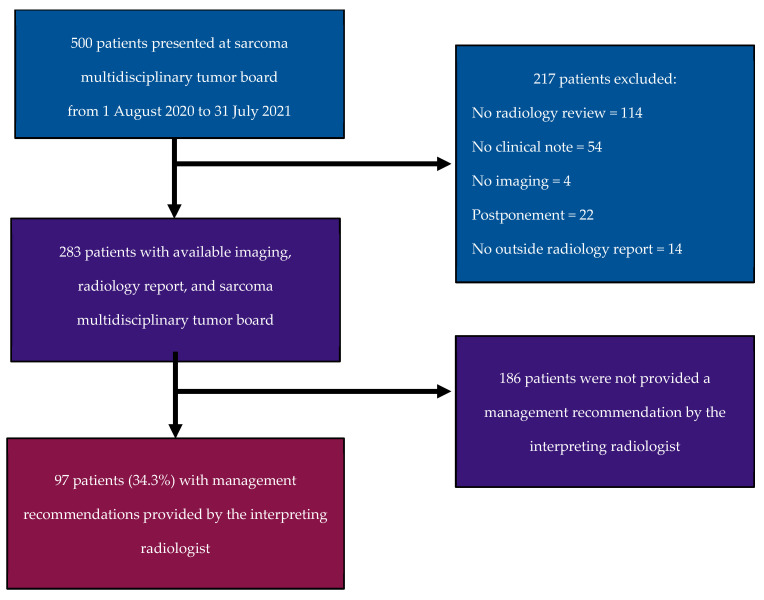
Flowchart of patient enrollment, exclusion criteria, and final study population.

This institutional review board (IRB)-approved, Health Insurance Portability and Accountability Act (HIPAA)-compliant retrospective study was performed at a single university-based tertiary sarcoma referral center. The requirement for informed consent was waived. Our electronic health record (EHR) system was queried for cases with a presumptive, MTB consensus-based, or established histopathologic diagnosis of soft tissue sarcoma who were presented at our weekly soft tissue sarcoma MTB from 1 August 2020 to 31 July 2021. Our weekly MTB is an hourly meeting in which 15–20 patients with sarcomas are discussed, approximately 5–10 of which are presented for imaging review. Our MTB is composed of multiple sarcoma specialists, including medical oncologists, radiation oncologists, pathologists, surgical oncologists, and radiologists. A consecutive series of 500 retrieved cases was assessed. Cases presented at our soft tissue sarcoma MTB for which imaging review was requested, with radiology reporting and MTB disposition available on EHR, were included. Patients were excluded for (1) the absence of a radiology review request, (2) the absence of clinical documentation of the MTB discussion and plan, (3) the absence of an imaging study in our institutional Picture Archiving and Communication System, (4) the postponement of patient presentation at the MTB, or (5) the absence of a radiology report (Figure 1). 

### 2.2. Data Collection

The data collected are summarized in Table 1 and included the type of imaging study reviewed, the patient’s clinical status, the origin of the radiographic imaging report (whether reported in-house or at an outside facility), the response to treatment assessment based on the semantic analysis of the radiology reports and of the MTB summary notes, the diagnostic and therapeutic recommendations present on the radiology reports and present on the MTB summary notes, and whether the radiology report’s recommendation was followed by the referring provider.

A review of the radiology reports was performed for each imaging study in query by the MTB. If multiple radiographic studies were available, all relevant reports were reviewed. Clinical data (patient clinical status, response assessment and recommendation by the MTB based on disposition documented in the EHR) were collected by two co-authors (A.C.R., V.P.), whereas radiologic data (the type of imaging study in review by the MTB, the response assessment on the radiology reports, and the recommendation on the queried radiology reports and whether the radiology report’s recommendation was followed by the referring provider) were collected by two radiologists in training (M.J., J.J.V.). All collected data were reviewed by a fellowship-trained radiologist (F.A.) with at least 5 years of experience in cancer imaging.

### 2.3. Statistical Analysis

Categorical variables were summarized using descriptive statistics such as frequencies and percentages. The agreement between the initial interpreting radiologist and the sarcoma MTB’s diagnostic assessments and clinical recommendations was calculated using Cohen’s kappa statistic and the percent agreement. For Cohen’s kappa, values of 0.01–0.20 were interpreted as no agreement, 0.21–0.39 as minimal agreement, 0.40– 0.59 as weak agreement, 0.60–0.79 as moderate agreement, 0.80–0.90 as strong agreement, and >0.90 as almost perfect agreement [20]. The McNemar–Bowker test was used to compare paired proportions, with Bonferroni adjustment to correct for multiple testing and a Z test to compare nonpaired proportions. If multiple studies were in query, the agreement with the MTB was evaluated only if all reports were concordant amongst themselves in terms of disease assessment and management recommendations. A *p* value < 0.05 based on a two-sided hypothesis was indicative of a statistically significant difference. Analyses were conducted using statistical software (JMP^®^, Version 15.0.0 SAS Institute Inc., Cary, NC, USA, 1989–2023).

## 3. Results

### 3.1. Study Population

A total of 283 sarcoma tumor board cases were eligible following the exclusion of 217 cases (Figure 1). Of the 283 cases included, 175 (61.8%) patients were on treatment, 86 (30.4%) were on surveillance, and 22 (7.8%) cases were neither (14 newly diagnosed soft tissue sarcomas, eight with conditions different from soft tissue sarcoma). The most common imaging studies reviewed were CT (157/283, 55.5%), followed by MRI (50/283, 17.7%) and PET/CT (36/283, 12.7%). In 40/283 cases (14.1%), multiple imaging modalities were reviewed. A total of 182 (64.3%) studies were reported in-house, while 101 (35.7%) studies were initially reported at outside facilities (Table 2). 

### 3.2. Disease Assessment 

**Figure 2:**
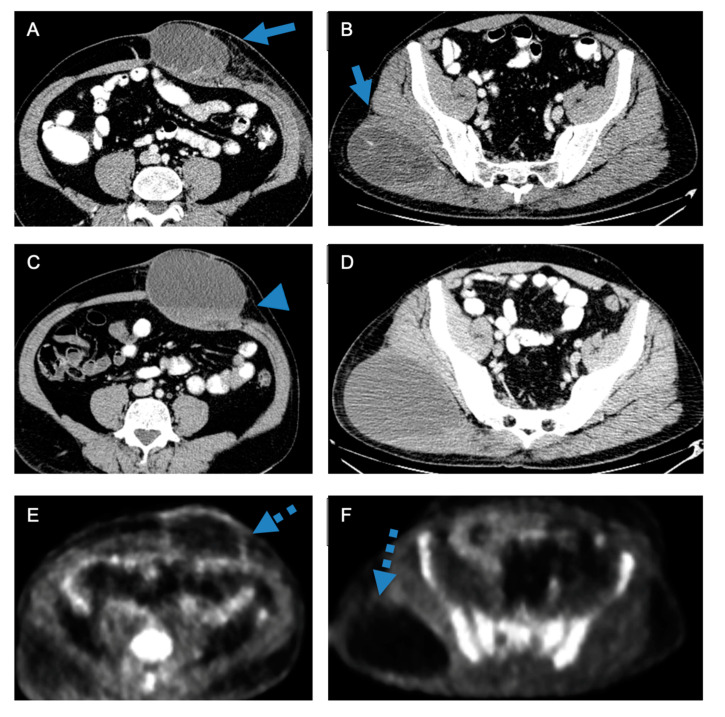
Discrepancy between radiology report and multidisciplinary tumor board assessment. A 55-year-old man with a metastatic high-grade undifferentiated pleomorphic sarcoma. Axial CT images (**A**,**B**) obtained at the time of diagnosis show two hypodense masses in the right posterior gluteal region and in the anterior abdominal wall (arrows). Axial CT images (**C**,**D**) obtained after 4 cycles of gemcitabine/taxotere show the mildly increased size of the masses but a decreased internal density and areas of fluid–fluid level within the anterior abdominal wall mass (arrowhead). The radiology report suggested the progression of the disease given the increase in the size of the lesions. The case was reviewed at a multidisciplinary tumor board and the consensus was that the increased tumor size was likely due to increased necrosis and possibly internal bleeding, suggesting a response to treatment. The findings were interpreted as stable disease. The patient was kept on gemcitabine/taxotere. Follow-up PET images (**E**,**F**) show only mild peripheral FDG uptake within the two masses (dashed arrows), suggesting a response to treatment.

A total of 35/283 cases were excluded from the disease assessment, as either cancer-naïve/lack of prior imaging for comparison (*n* = 22) or due to new disease (*n* = 13). Among the 248 cases for which disease assessment was evaluated, there was moderate agreement on the disease response between the MTB and radiology reports, where 214/248 were concordant (86.2% agreement; κ = 0.78, *p* value < 0.0001). Radiologists interpreted progressive disease/recurrence significantly more commonly than MTBs (54.4% vs. 44.4%, Bowker’s–McNemar test *p* < 0.001) but stable disease significantly less commonly than MTBs (28.2% vs. 36.3%, Bowker’s–McNemar test *p* < 0.001) (Table 3) (Figure 2) (Figure S1). 

No difference was identified in the radiologists’ and MTBs’ interpretations for partial response (17.3% vs. 19.4%, Bowker’s–McNemar test *p* = 0.17). No cases were interpreted as a complete response by the radiologists or the MTBs.

When comparing the disease assessment by the MTB versus radiology reports stratified by disease status, there was moderate agreement (84.3% agreement; κ = 0.76, *p* value < 0.0001) amongst 166 patients undergoing treatment at the time of the MTB and strong agreement (90.2% agreement; κ = 0.81, *p* value < 0.0001) amongst 82 patients undergoing surveillance. 

When the disease assessment by the MTB versus radiology reports was stratified by modality (CT vs. MR vs. PET/CT vs. multiple imaging studies), the agreement was highest for MR (88.1% agreement, κ = 0.79, *p* value < 0.0001), followed by CT (86.3% agreement, κ = 0.78, *p* value < 0.0001) and PET/CT (82.4% agreement, κ = 0.71, *p* value < 0.0001). There was moderate agreement in cases with multiple imaging modalities (87.9% agreement, κ = 0.79, *p* value < 0.0001).

When the disease assessment by the MTB versus radiology reports was stratified by location (in-house vs. outside reports), the agreement was slightly higher for outside reports (in-house reports: 83.9% agreement, κ = 0.74, *p* value < 0.0001; outside reports: 90.7% agreement, κ = 0.85, *p* value < 0.0001), although the proportion of agreement was not significantly different (z = −1.4703, *p* value = 0.14).

### 3.3. Radiology Recommendations

Only 97/283 reports (34%) contained a clinical recommendation. When the reports containing a clinical recommendation were compared with the MTB recommendation, there was no agreement between the radiology reports and MTB (36% agreement, κ = 0.18, *p* value < 0.0001).

The most common radiology recommendations were short-term follow-up and regular follow-up based on the surveillance imaging protocol (Table 4) (Figure S2).

MTBs most frequently recommended a tissue biopsy or surgical, radiation oncology, an interventional radiology referral, regular oncologic follow-up, or a change in systemic therapy. Of the 97 recommendations present on the radiology reports, 71 were carried out by the referring provider (73%), of which short-interval and regular oncologic follow-up were the most commonly followed recommendations (both 28/71, 39%) (Table 4).

Radiologists recommended short-term follow-up or additional imaging more commonly than MTBs (46.4% vs. 21.7%, Bowker’s–McNemar test *p* < 0.001) but tissue biopsies or surgical, radiation oncology or interventional radiology referrals less commonly than MTBs (12/97, 12.4% vs. 33/97, 34%, Bowker’s–McNemar test *p* = 0.002) (Table 4). No difference was identified in radiologists’ and MTBs’ recommendation of regular follow-up (32% vs. 26.8%, Bowker’s–McNemar test *p* = 0.35). No radiology reports recommended the initiation of or a change in systemic therapy, whereas no MTB provided “other” recommendations.

When the recommendations by MTBs versus radiology reports were stratified by modality (CT vs. MR vs. PET/CT vs. multiple imaging studies), we found no agreement (CT: 35.8% agreement, κ = 0.2, *p* value = 0.31; MR: 21.4% agreement, κ = 0.14, *p* value = 0.35; PET/CT: 25% agreement, κ = 0.11, *p* value = 0.71; multiple imaging modalities: 38.9% agreement, κ = 0.2, *p* value = 0.76).

When the recommendations by the MTBs versus radiology reports were stratified by location (in-house vs. outside reports), the agreement was slightly higher for outside reports (in-house reports 35.7% agreement, κ = 0.16, *p* value = 0.005; outside reports: 40.7% agreement, κ = 0.23, *p* value = 0.01), although the proportion of agreement was not significantly different (z = −0.4593, *p* value = 0.65).

When the disease assessment was stratified by the presence or absence of recommendations in the radiology report, the agreement was significantly lower for cases in which a recommendation was present (9.4% agreement, κ = 0.67, *p* value < 0.0001), compared to cases in which recommendations were not provided (82.9% agreement, κ = 0.83, *p* value < 0.0001) (z = −2.1099. *p* = 0.03).

## 4. Discussion

In this study, we investigated the agreement between standard clinical radiology reports from academic and non-academic centers and MTBs in terms of the radiological assessment of the disease, response to treatment, and clinical recommendations. We showed that there was moderate agreement on disease assessment between the MTB and radiology reports and that the clinical recommendations provided by the MTB and the radiology reports often differ. In fact, MTBs reached different conclusions from radiology reports 17.7% of the time in terms of disease assessment and provided different clinical recommendations 64% of the time.

We found moderate agreement between the MTBs and radiology reports on disease assessment (86.2% agreement; κ = 0.78). A study by Zhang et al. on 343 patients with liver tumors showed that 309 patients were presented to the tumor board with a clinical diagnosis from an outside provider. The clinical diagnosis was altered in 26/309 (8.4%) following review by the MTB. Of the cohort that received a change in clinical diagnosis, 17/26 (65.4%) resulted from a change in imaging interpretation by the MTB [21]. Highlighting the importance of imaging findings in guiding decision-making, Lamb et al. reported inadequate radiologic information as the most common impediment to the generation of management recommendations in MTBs [22].

Radiologists were 11% more likely to assign progressive disease/recurrence than MTBs (54.4% vs. 44.4%; *p* value < 0.001). Campani et al. highlighted similar findings in a study evaluating 125 patients with HCC on atezolizumab–bevacizumab. The study illustrated a noticeable difference in patients classified as having progressive disease after radiologic review with the Response Evaluation Criteria in Solid Tumors (RECIST) and modified RECIST (mRECIST) criteria, who were later classified as stable or responding to treatment by the MTB. The discrepancies between the interpreting radiologists and the MTB were most often due to interval increases in size being misinterpreted as progressive disease by the radiologists [23]. This may be due to the lack of clarity and standardization of the definition of progressive disease in radiology reports. Although tumor response criteria such as RECIST offer clear guidelines and definitions of what constitutes progressive disease, they are not routinely used in clinical care and not used in our practice for clinical reads [24,25]. As such, the assessment of the treatment response relies on the subjective evaluation of the radiologist, leading to the potential misinterpretation of progressive disease, and this may explain why radiologists interpreted progressive disease/recurrence significantly more commonly than MTBs, with 21/248 cases categorized as stable disease by the MTB but misinterpreted as progressive disease/recurrence by radiologists. Although it is unclear whether the implementation of the standardized reporting of the oncologic response may decrease the discrepancies in the disease assessment between radiologists and MTBs, a recent study comparing the assessment of progressive disease between routine clinical reads and RECIST 1.1 assessments showed that 105/327 (32%) of cases labeled as stable disease on the RECIST 1.1 assessment were overdiagnosed as PD on clinical reads, suggesting that standardized reporting may decrease the discrepancies in the disease assessment between radiologists and MTBs [25,26].

When the disease assessment by the MTB versus radiology reports was stratified by imaging modality, moderate agreement was appreciated across imaging modalities; however, it was highest for MR (88.1% agreement, κ = 0.79). This may be related to the fact that, in our practice, while MRI is reported by radiology subspecialists, highly experienced in the imaging of soft-tissue sarcoma, CT is reported either by general radiologists or cancer imaging subspecialists. This may potentially be a contributing factor to the discrepancies in disease assessment between radiologists and MTBs: while oncology radiologists are generally familiar with the concept of pseudoprogression (i.e., an increase in the size of a tumor related to the response to treatment or edema), general radiologists may misinterpret pseudoprogression as progressive disease. A study by Briggs et al., assessing the discrepancy rate between general radiologist and subspecialist neuroradiologist reports, revealed that there was only 66% agreement between specialists and general radiologists, with no significant difference between imaging modalities [27].

Of the 283 included radiology reports, 97 included clinical recommendations. Overall, there was no agreement between the radiology recommendations and MTB recommendations (κ = 0.18, *p* value < 0.0001). Despite the lack of agreement, the majority (71/97; 73.1%) of the recommendations provided by radiologists were followed by the ordering providers. It is unclear why this happened, and we can hypothesize that the recommendations were followed before the cases were presented at our weekly MTB. Approximately 15–20 cases are presented to our MTB weekly, with only 5–10 cases for radiology review; many cases are presented weeks after imaging has been obtained, which may have led to the providers following the recommendations before this was presented to the MTB, especially if these recommendations are perceived as “urgent” by the referring providers and if the referring providers are not sarcoma specialists. Furthermore, a lack of action from the provider after a recommendation is given by the radiologist may be perceived by the referring provider as having legal implications. Nonetheless, since we did not investigate why the recommendations were followed, our hypotheses are purely speculative. A single-center study by White et al. on 598 radiology reports and the taxonomy of recommended actions and time frames included in reports demonstrated that 87.4% of radiology recommendations were carried out by the ordering provider—similar to the results achieved in this study [19]. When expanded to multiple network hospitals, however, as depicted in a study by Mabotuwana et al. on 2,972,164 radiology examinations and an evaluation of only imaging recommendations, the overall implementation was 58.1% [18].

While MTBs more often recommended a tissue biopsy or surgical, radiation oncology or interventional radiology referrals relative to radiologists, radiologists more often recommended short-interval follow-up or additional imaging (46.4% vs. 21.7%, *p* < 0.001). Similarly, White et al., in a sample of 598 radiology reports, identified that the most common recommendations were to obtain additional imaging (77.3%), whereas specialty referrals (19.9%) and invasive image-guided procedures (7.8%) were less common [19]. This may reflect that radiologists may be reluctant to recommend a tissue biopsy or surgical, radiation oncology or interventional radiology referrals given the high number of equivocal findings encountered on imaging studies, or that those referrals made by the MTB may be recommended for reasons unknown to the radiologists when reading imaging studies, although this is purely speculative.

We found that radiology reports with recommendations had significantly lower agreement on the disease assessment compared with radiology reports with no recommendations (74.2% vs. 86.6% agreement, *p* value = 0.01). We can speculate that an incorrect assessment of the disease will lead an ”incorrect” recommendation, although it is unclear how discrepancies in disease assessment may contribute to differences in management recommendations.

Limitations that warrant consideration include the low number of cases and the heterogeneity of soft tissue sarcoma, which may alter the generalizability of the findings. Additionally, the study was conducted through a chart review, requiring inference and the interpretation of clinical notes, potentially introducing inaccuracies and variability in the data interpretation. Furthermore, the discrepancies may have been partially related to inter-reader variability, given the partial overlap between the clinical radiologist and the subspecialized radiologists covering the tumor board. Furthermore, we did not assess how many of the interpreting radiologists were subspecialists versus general radiologists, which may have had an impact on the accuracy of the disease assessment and the validity of the recommendations. We did not collect data on the extent of the tumor burden for patients on treatment with metastatic disease. As such, we do not know if the extent of the disease affected the radiologists’ disease assessment and ultimately impacted the discrepancies. Finally, we did not perform a qualitative analysis of the discrepancies in the disease assessment and recommendations between MTBs and radiology reports. 

Further research with larger sample sizes and standardized methodologies, as well as prospective studies investigating the correlation between MTB disease assessments and treatment outcomes, may strengthen the power and improve the applicability of these findings. While this study serves as a pilot for future endeavors, further investigation between radiologists involved in MTBs and those who provide care more peripherally should be explored to see how MTB involvement influences the concordance between the interpreting radiologist and MTB consensus. 

This study highlights the potential pitfalls that can occur when relying solely on radiology reports to direct sarcoma patient care, which is inherently prone to errors in diagnosis [28]. The findings revealed that patient care driven by radiologic evidence has its limitations, as approximately 18% of the interpretations made by radiologists were discordant with the MTB in terms of disease assessment. The MTB often has pertinent clinical data that radiologists do not have immediately available during their interpretation. This discrepancy emphasizes the importance of collaborative decision-making processes, involving input from various specialties, to ensure comprehensive and accurate sarcoma patient management.

Incorporating radiology interpretations into MTB assessment has been shown to impact patient care in the management of various malignancies [11,12,13,14,15]. Our study also highlights that while radiologists play a valuable role in assessing sarcoma disease, their recommendations often diverge from those of the MTB, emphasizing the need for improved concordance. In an effort to address this, it is recommended that radiologists actively engage with MTBs to gain insight from diverse clinical perspectives prior to finalizing a radiology report, whenever possible. As such, radiologists should familiarize themselves with the best clinical practices to ultimately improve the concordance of the disease response and recommendations. This will ultimately enhance the overall quality of patient care in the context of sarcoma management and could broadly be applied to other cancers alike. Key challenges in incorporating multidisciplinary care in routine radiology practice include time and effort, the loss of productivity, logistics and coordination, burnout, and occasional conflicting interpretations that may undermine consensus-building on the tumor board [29,30,31,32].

## 5. Conclusions

The role of radiologists in the MTB setting is valuable in assessing disease in patients with sarcoma; however, radiologists’ recommendations are often incongruent with the MTB’s consensus, as demonstrated in this study. This study has highlighted the discordance and encourages all radiologists, whether in an academic or private practice setting, caring for patients with sarcoma to participate in MTBs to best align their interpretations with optimal clinical management and make multidisciplinary aligned recommendations whenever feasible. Further research should investigate the influence that participation in MTBs has on the interpreting radiologist’s assessment of the disease state and the recommendations provided. Given the challenges in incorporating MTBs into day-to-day radiology workflows, further research into workflow models could be of value.

## Figures and Tables

**Table 1:** Data collection survey performed on both radiology reports and sarcoma tumor board responses.

Imaging Modality	Computed Tomography (CT) Magnetic Resonance Imaging (MRI) Positron Emitting Tomography/CT (PET/CT) Multiple Imaging Modalities
In-House vs. Outside Interpretation	In-House Report Outside Report
Patient’s Clinical Status	On Treatment On Surveillance New Diagnosis
Response Assessment	Stable Disease Partial Response Complete Response Progressive Disease/Recurrence
Recommendation	No recommendation issued Tissue biopsy or surgical/radiation oncology/interventional radiology referral Short-term follow-up/additional imaging needed Regular follow-up as per oncologic protocol Initiation of or change in systemic therapy Other (clinical correlation)
Adherence to Radiologist Recommendation	Yes No

**Table 2:** Clinical status and imaging modalities of the study population.

Imaging Modality	Computed Tomography (CT): 157 (55.5%) Magnetic Resonance Imaging (MRI): 50 (17.7%) Positron Emitting Tomography (PET/CT): 36 (12.7%) Multiple Imaging Modalities: 40 (14.1%)
Patient’s Clinical Status	On Treatment: 175 (61.8%) On Surveillance: 86 (30.4%) Neither/New Diagnosis: 22 (7.8%)
In-House vs. Outside Interpretation	In-House Report: 180 (63.6%) Outside Report: 103 (36.4%)

**Table 3:** Multidisciplinary tumor board disease assessment and clinical radiologist disease assessment.

	Radiologist Disease Assessment					
**Multidisciplinary Tumor** **Board Assessment**		**CR**	**SD**	**PR**	**PD**	**Total**
	**CR**	0	0	0	0	0
	**SD**	0	68	1	21	90
	**PR**	0	2	39	7	48
	**PD**	0	0	3	107	110
	**Total**	0	70	43	135	248

SD: stable disease; PR: partial response; CR: complete response; PD: progressive disease/recurrence.

**Table 4:** Comparison of clinical radiologists’ recommendations and multidisciplinary tumor board’s recommendations.

	Radiologist Recommendations						
**Multidisciplinary Tumor** **Board Recommendations**		**Bx**	**SFU**	**RFU**	**Tx**	**Other**	**Total**
	**Bx**	5	12	9	0	7	33
	**SFU**	2	17	1	0	1	21
	**RFU**	1	10	14	0	1	26
	**Tx**	4	6	7	0	0	17
	**Other**	0	0	0	0	0	0
	**Total**	12	45	31	0	9	97

Bx: tissue biopsy or surgical/radiation oncology/interventional radiology referral; SFU: short-term follow-up/additional imaging needed; RFU: regular follow-up as per oncologic protocol; Tx: initiation or change in systemic therapy; other: other recommendations, including clinical correlation.

## Data Availability

Data are unavailable due to ethical restrictions.

## Supplementary Materials

The following supporting information can be downloaded at: https://www.mdpi.com/article/10.3390/cancers16152674/s1, Figure S1: Sankey diagram of radiologists’ and multidisciplinary tumor board’s disease assessment. Figure S2: Sankey diagram of radiologists’ and multidisciplinary tumor board’s recommendations.
