# Supplementary figures and images for: Imaging-Based Disease Assessment and Management Recommendations: Impact of Multidisciplinary Sarcoma Tumor Board
Source: Cancers (Basel). 2024 Jul 26;16(15):2674. doi: 10.3390/cancers16152674 (PMC11311895; doi:10.3390/cancers16152674)

## Slide 1
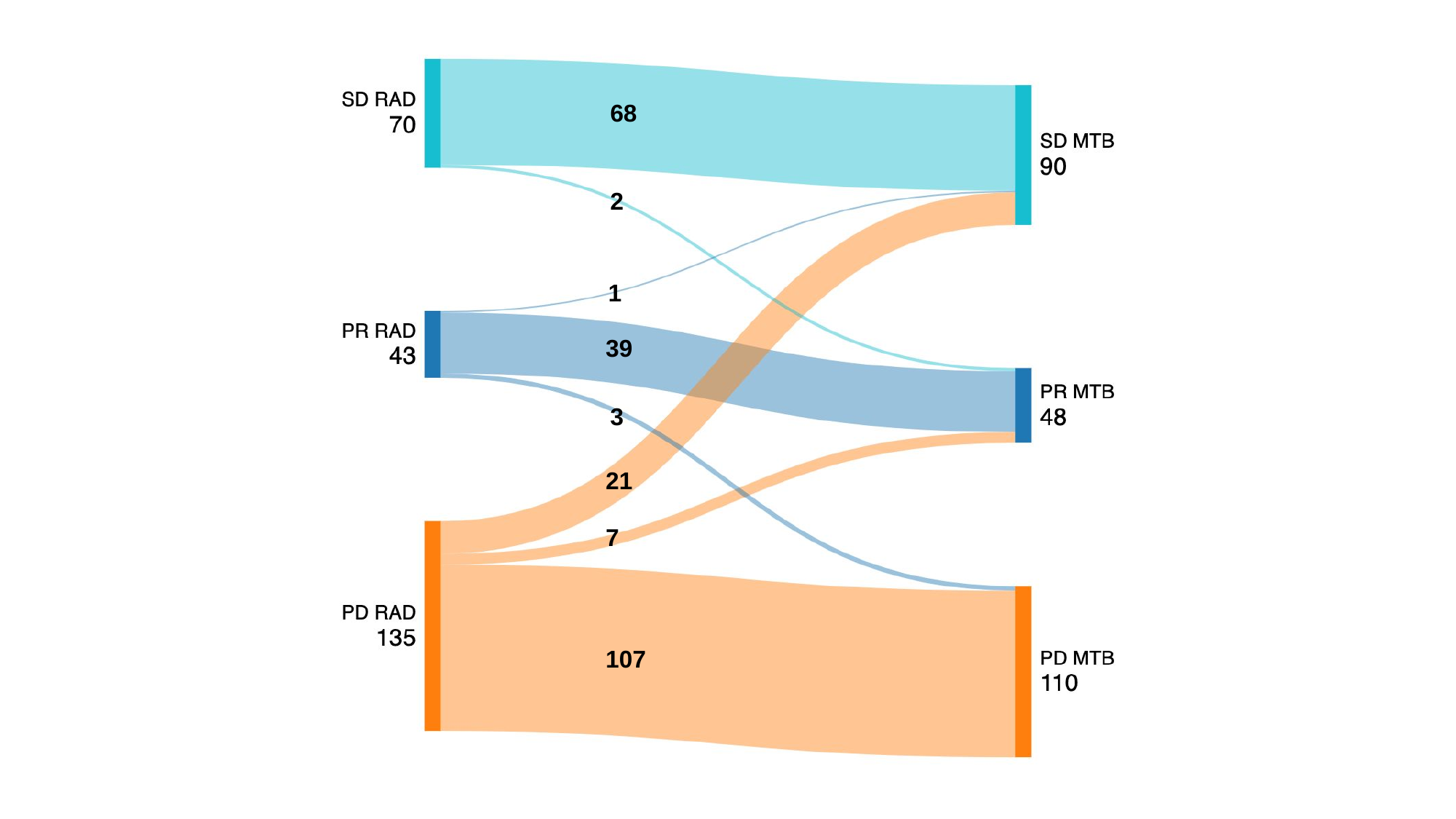

68
2
1
39
3
107
21
7

## Slide 2
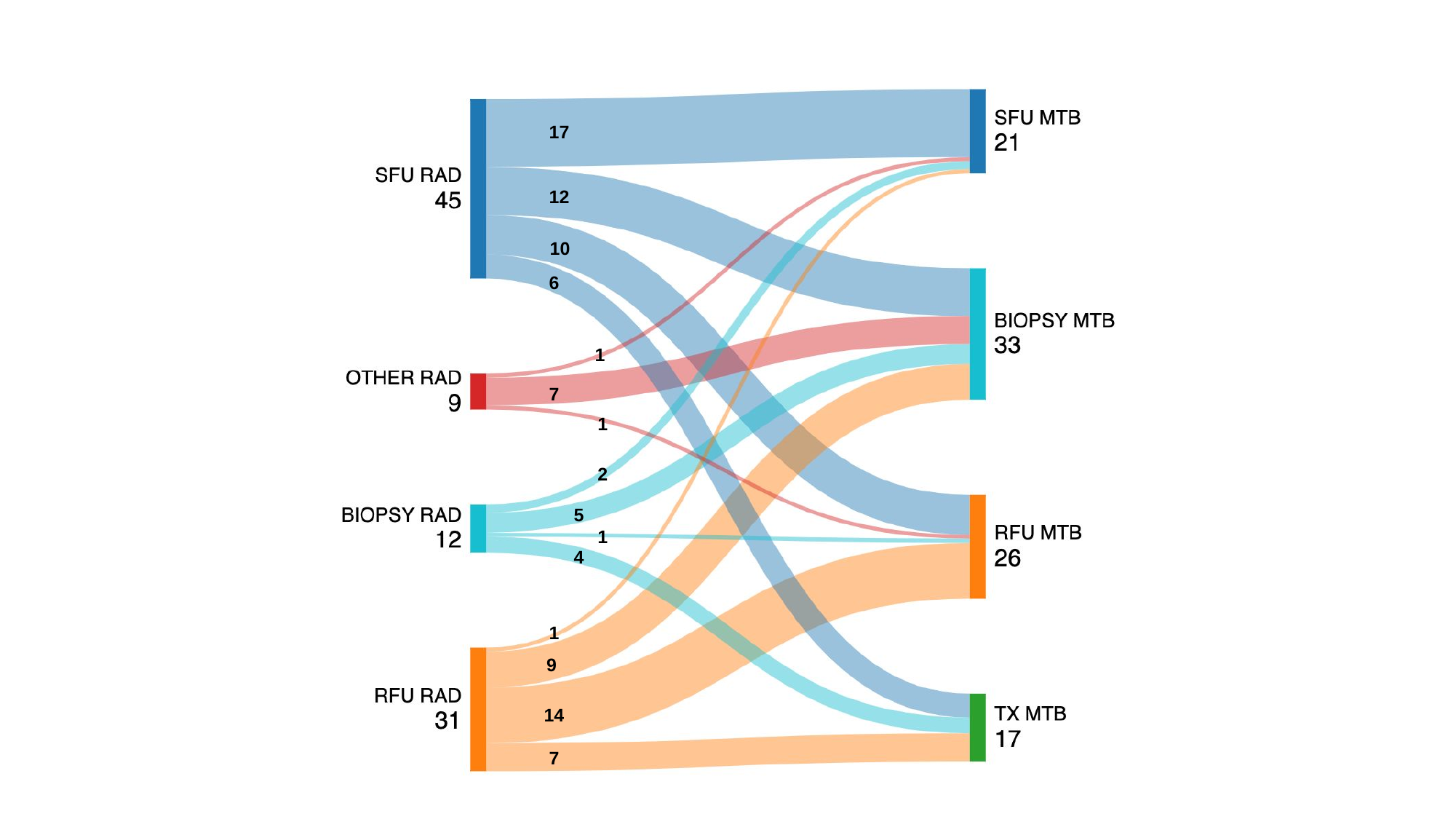

17
12
10
6
1
7
1
2
5
1
4
1
9
14
7

Supplement: Supplementary file 1 [file cancers-16-02674-s001.zip › cancers-3090731-supplementary.pptx]
